# Basic Amino Acids as Salt Substitutes in Low-Salt Gel-Based Meat Products: A Comprehensive Review of Mechanisms, Benefits, and Future Perspectives

**DOI:** 10.3390/foods14040637

**Published:** 2025-02-14

**Authors:** Chuanlong Yu, Wenbing Hu, Lingli Chen, Kehui Ouyang, Hui Chen, Suyun Lin, Wenjun Wang

**Affiliations:** 1 College of Food Science and Engineering, Jiangxi Agricultural University, Nanchang 330045, China; yuchuanlongvip@163.com (C.Y.);; 2 College of Animal Science and Technology, Jiangxi Agricultural University, Nanchang 330045, China

**Keywords:** salt reduction, basic amino acids, protein properties

## Abstract

Gel-based meat products have appealing market potential due to their unique texture, elasticity, and tender taste. Sodium chloride (NaCl) is commonly used in these products to enhance flavor, improve texture, ensure food safety, and extend shelf life. However, excessive long-term NaCl intake is connected with health issues such as hypertension and cardiovascular diseases, raising concerns about its impact on human health. As a result, the reduction of NaCl in these products, while maintaining their flavor and texture, has become a key area in the food industry. Salt reduction strategies often compromise product quality, limiting the search for substitutes. Consequently, there is growing interest in developing new salt substitutes. Recently, basic amino acids (BAA) have emerged as a viable alternative to NaCl in low-salt gel-based meat products. Studies have shown that BAAs not only enhance the solubility, gelation, and emulsification properties of salt-soluble proteins but also reduce protein and lipid oxidation in low-salt conditions, improving sensory characteristics and texture. When combined with chloride salts, BAAs can further lower salt content while improving the quality of the products. In addition, adding modern processing techniques (such as ultrasound, pulsed electric fields) has indicated positive effects on the taste and texture of low-salt meat products. Future studies should deploy advanced tools to dissect the micro-/macro-level impacts of BAAs on low-salt gel products. Furthermore, integrating modern food processing and information technologies could lead to the development of personalized, intelligent low-salt meat products that satisfy consumer demands for both health and taste.

## 1. Introduction

With the improvement of global health levels, it is projected that approximately two billion people will lead healthier lives by 2030 [1], posing new challenges for the provision of rich, healthy, and sustainable nutrition. Animal proteins such as beef and fish, being rich in essential amino acids, have become crucial nutritional sources to support human health. The amino acid composition of meat proteins resembles the growth and maintenance requirements of human tissues, facilitating human growth and sustaining life activities. Meat is an important source of protein for humans. The proteins in it play a positive role in promoting bone development, enhancing cell activity, and repairing body tissues, which are essential for maintaining good health [2,3]. Interestingly, gel-based meat products, as an important category in meat processing, have attracted a large number of consumers with their unique elasticity, toughness, and tender taste. Gel-based meat products take meats like livestock, poultry, and fish as main materials. With additives such as NaCl, phosphates, starch, and protein additives, plus chopping, emulsifying, and heating, the myofibrillar proteins (MPs) and myosin in the meat are made to denature and aggregate, thus forming a meat product with a 3D gel network structure. Typical ones are sausages (Chinese sausages and Western sausages), hams (Chinese hams and Western hams), luncheon meats, and meatballs (pork balls, beef balls, and fish balls). Numerous studies have shown that these unique sensory characteristics are mainly attributed to the gelation properties of salt-soluble proteins (such as myosin). Myosin is a protein with a special structure, consisting of two hydrophobic globular heads and a hydrophilic long helical tail. The head is responsible for binding to adenosine triphosphate (ATP) and conducting hydrolysis reactions, while the tail helps to form polymeric structures with other myosin molecules. At a low salt concentration (<0.3 mol/L), myosin typically forms a network structure that is characterized by porosity and discontinuity; whereas at a relatively high salt concentration (≥0.3 mol/L), the interactions between myosin are separated due to electrostatic shielding, thus improving the gel structure and the palatability of meat products.

During the processing of gel-based meat products, the role of NaCl is indeed worthy of recognition. However, the negative impacts of long-term high-salt diets on health cannot be overlooked. In most countries and regions, the daily salt consumption per person is approximately 9–12 g, exceeding the 5 g per day recommended by the WHO [4]. Long-term high-salt diets pose risks to human health, such as heart diseases and hypertension [5]. Owing to the detrimental health consequences of high-sodium diets, numerous countries across the globe have formulated salt reduction strategies. In the UK, the daily NaCl intake declined from 9.5 g in 2005 to 8.1 g in 2012 [6]. Additionally, the “Healthy China 2030” Plan Outline of China aims to decrease the daily NaCl intake per capita by 20% by the year 2030 [7].

In recent years, the use of BAAs, represented by L-Lys, L-His, and L-Arg in meat products, has attracted extensive attention. Due to their unique chemical structures, L-Lys, L-His, and L-Arg can interact with salt-soluble proteins, altering intermolecular forces such as ionic and hydrogen bonds, thereby enhancing protein solubility, suppressing excessive aggregation, and improving the quality of gel-based meat products. L-Lys has an ε-amino group that is prone to ionization, conferring to it a strong alkalinity; the guanidine group of L-Arg carries a positive charge under all conditions, playing a particularly significant role in its interaction with proteins; L-His can provide proton donors and acceptors, bringing with it a certain antioxidant capacity [8]. Interestingly, BAAs could increase the gelation performance of meat products by modifying the structure of myosin and increasing its solubility under low-salt conditions. Consequently, L-Lys, L-His, and L-Arg have been widely utilized in the low-salt modification of meat products such as ham [9,10]. Existing studies have mainly focused on the observable effects of BAAs on meat product quality, such as the improvement of gelation performance and the reduction of oxidation [11,12]. However, despite the growing research on BAAs in meat products, there are still substantial research gaps. Our understanding at the molecular level remains severely limited. We merely have a basic understanding of the overall interaction between BAAs and salt-soluble proteins. The initial binding sites of BAAs on these proteins are unknown, and when BAAs interact with key proteins like myosin, the exact sequence of interaction, whether starting from the hydrophobic heads or hydrophilic tails and whether intermolecular force modification occurs simultaneously or step-by-step, remains unclear. This lack of knowledge restricts the development of better modification strategies for low-salt gel-based meat products. It is not well-defined how the changes in intermolecular forces precisely translate into the improvement of protein solubility and gelation properties. Also, most research has been conducted under laboratory-controlled conditions, and there is a lack of studies on the application of BAAs in the large-scale industrial production of low-salt gel-based meat products. The impact of different processing parameters, such as temperature, time, and pH, on the effectiveness of BAAs in real-world production settings has not been comprehensively explored.

Given the aforementioned research voids, there is an immediate need to comprehensively and systematically summarize the mechanisms underlying the impact of BAAs on salt-soluble proteins under low-salt conditions. Thus, this paper aims to summarize the application of BAAs in low-salt gel-based meat products, explore the potential mechanisms by which they improve the quality of low-salt gel-based meat products, and discuss the challenges associated with using BAAs as substitutes for NaCl. Additionally, this paper also provides suggestions and prospects for future research directions.

## 2. The Role of NaCl in Traditional Meat Products

The color, aroma, and taste of food are significant factors influencing consumers’ purchasing decisions and eating experiences. Among them, NaCl is a crucial condiment for adjusting the flavor and taste of food and is vital to meat product processing.

**Figure 1:**
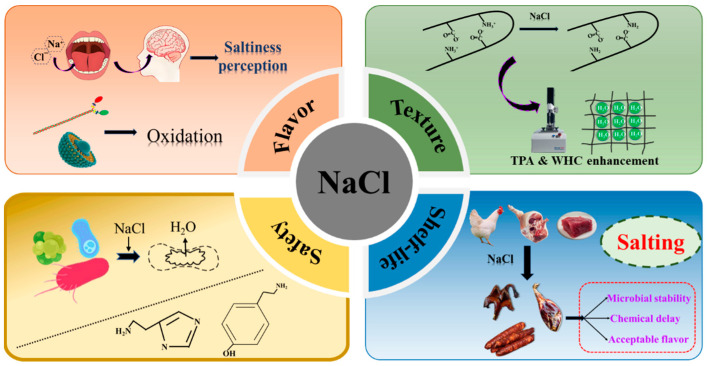
The role of NaCl in gel-based meat products.

As shown in Figure 1, the roles of NaCl in the processing of meat products can be specifically summarized into the following four main aspects: (1) Improving flavor. One of the basic roles of NaCl in meat products is to enhance their flavor. In meat products, NaCl mainly enables the perception of saltiness through the regulation of taste by Na^+^ and Cl^−^ [13]. Once meat products enter the oral cavity, the interaction with saliva during chewing causes the migration of Na^+^ to the surface of the tongue and activates the sodium-sensing channels within the mouth, thus triggering the saltiness sensation [14]. Moreover, the significance of NaCl in the flavor creation of meat products is also influenced by multiple factors, such as the type of meat and processing conditions. Protein and lipid oxidation also play significant contributions in shaping the taste profile of low-salt meat products. The variation in NaCl concentration is closely related to the oxidation levels of proteins and lipids in meat products. Gan et al. [15] found that in the making of traditional cured pork, replacing 70% of NaCl with KCl could facilitate protein degradation and oxidation, leading to a reduction in tryptophan fluorescence and an increase in the total carbonyl. Reducing the NaCl content promotes protein hydrolysis and oxidation, thereby increasing free amino acid content. Conversely, other researchers studied the effects of NaCl on protein oxidation and lipid oxidation in Chinese dry sausages with salt contents of 2% and 4% (*w*/*w*); they found that a 4% NaCl concentration could promote protein oxidation and lipid hydrolysis, and there was a significant relationship between protein and lipid oxidation [16]. (2) Enhancing texture. NaCl is also vital for the texture improvement of meat products. In processed meats, 0.6 M NaCl can promote the solubilization of myosin and improve hydration and water-holding capacity (WHC), thus improving gel characteristics [17]. By interacting with the positively charged regions of salt-soluble proteins, NaCl can neutralize these charges and disrupt salt bridges, leading to the separation of muscle fibers and enhanced hydration and further improving the texture of meat products [18]. However, some studies have also pointed out that NaCl contributes to the creation of salt bridges, while hydration is mainly caused by non-electrolyte substances [19]. Moreover, NaCl enhances the formation of disulfide bonds between chicken breast myoprotein, further increasing hydration and gel strength, reducing cooking loss, and improving the viscosity of chicken products, which helps to improve oral texture and sensory acceptability [20]. (3) Enhancing safety. NaCl is also central to improving the safety of meat products. By regulating osmotic pressure, NaCl can effectively lower the water activity (Aw), thereby inhibiting bacteria growth and reproduction. In addition, NaCl antibacterial action has two other mechanisms: causing osmotic shock to dehydrate microbial cells and interfering with intracellular enzyme activity to reduce oxygen solubility and make cells expend energy expelling Na^+^ [21]. Previous studies have found that there is a positive correlation between the NaCl content in meat products and its antibacterial effect [22,23,24]. Huang et al. [24] discovered that the flavor and main microbial communities in shrimp paste varied with different salt concentrations during fermentation. Similarly, in refrigerated conditions, biogenic amines in dry-cured tuna in the low-salt group were seven times higher after one month and 13 times higher after three months compared to the high-salt group [22]. (4) Extending shelf life. As a traditional food preservative, NaCl can effectively inhibit the reproduction of microorganisms via lowering Aw and is thus widely utilized to prolong shelf life. For instance, salt-baked foods and pickled foods achieve the purpose of extending shelf life through the addition of a large amount of NaCl during food processing. NaCl is often seen as a food preservative because it lowers Aw, inhibiting microbial growth and extending product shelf life [25]. It has been found that microbial populations (expressed as Log10 CFU/g) were consistently much higher in products with half the salt content, and the considerable growth of Pseudomonas spp. led to sensory characteristics obviously related to spoilage. A similar phenomenon was also observed in the study of pork sausages, where salt reduction (from 2.0% to 1.5%) could accelerate spoilage and shorten shelf life [26].

NaCl plays multifaceted and significant roles in the processing of meat products. It not only improves flavor, enhances texture, and increases safety but also extends shelf life. However, with the escalating demand for healthy diets, the reduction of salt intake has emerged as a crucial objective in global public health. The current research hotspot lies in how to reduce the utilization of NaCl without compromising the quality and safety of meat products.

## 3. The Effect of BAAs on Protein Processing Properties

**Figure 2:**
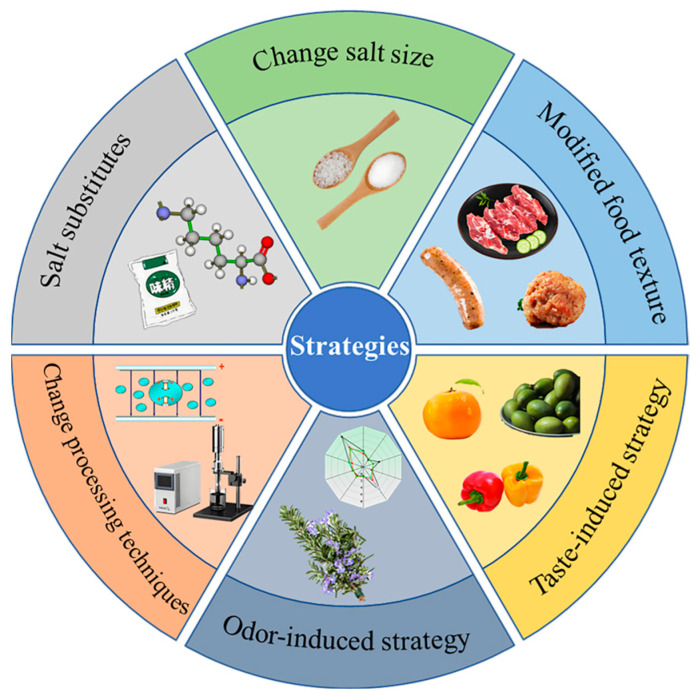
Salt reduction strategies in meat products.

The common strategies for salt reduction are diverse, each with its own advantages. As shown in Figure 2, the primary methods include directly reducing salt addition, substituting chloride salts, altering the form and particle size of salt, modifying food texture, combining technological processes, and enhancing the perception of saltiness through aroma–salt interactions.

The most direct approach to decreasing salt content in meat products is simply to lower the amount of NaCl. Typically, a moderate reduction in NaCl does not significantly affect the overall product quality. However, reducing NaCl content may prevent the adequate dissociation of salt-soluble proteins, which influences gel formation and decreases gel performance. To address this, the food industry has increasingly used KCl as a NaCl substitute, achieving some success in salt reduction. Studies have shown that when KCl replaces more than 50% of NaCl in products like smoked bass [27], low-salt ham [28], and pork [29], the result is not only a reduction in saltiness but also an increase in bitterness and a decline in textural properties. These findings indicate that a moderate substitution of NaCl with KCl can facilitate the unfolding of salt-soluble proteins, facilitating the formation of stable and irreversible gel networks through molecular forces such as disulfide bonds and hydrophobic interactions. However, excessive KCl substitution leads to protein aggregation, inhibiting crosslinking between salt-soluble proteins and consequently reducing WHC and gel properties. Many scholars have separately found that in smoked perch [27], low-salt ham [28], pork [29], and Alaskan pollock products [30], when the substitution amount of KCl exceeds 50%, it not only increases the bitterness and decreases the saltiness of the products but also reduces the texture properties of the products. In addition, the use of micro-particulate salt could lower the amount of salt required to reach the desired saltiness [31]. This is because microcrystalline salts dissolve more readily than larger particles, creating higher localized concentrations around taste receptors and enhancing the perception of saltiness. Moreover, the sausage recipe included 60% commercial surimi, 2% NaCl, 15% pork fat, 9% corn starch, 0.08% sodium erythorbate, 12.92% ice water, and 1% polysaccharides (carrageenan, gellan gum, xanthan gum, locust bean gum). These polysaccharides can modify the fish paste sausage’s gel structure, changing saltiness perception [32]. In recent years, novel processing technologies such as direct current magnetic fields [33], pulsed electric fields [34], ultrasound [35], microwaves [36], and high-pressure processing [37] have been increasingly applied to modify the microstructure of meat products, improve sodium–protein interactions, and enhance saltiness flavor. While these technologies offer the potential for the large-scale production of low-salt products, they increase the cost in the meat product industry.

**Figure 3:**
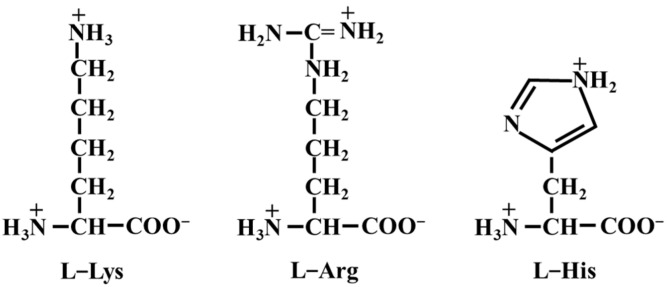
Structural formula of three basic amino acids.

Despite the progress made with current salt reduction strategies, challenges remain in maintaining saltiness perception while ensuring economic feasibility. Recent studies on the impact of BAAs, such as L-Lys, L-His, and L-Arg, on salt-soluble proteins and gel-based meat products have revealed that these amino acids can enhance protein solubility and improve gelation and emulsification properties [11,38,39]. These findings offer new insights for developing cost-effective and convenient salt reduction solutions. Consequently, they provide valuable scientific evidence for the meat industry in the search for efficient salt substitutes during the reduction process. Figure 3 reveals distinct chemical structures among three typical basic amino acids. Lysine is highly alkaline, arginine affects electrostatic interactions between proteins, and histidine improves the product’s antioxidant capacity [8]. Their applications in low-salt gel-based meat products are garnering widespread attention.

### 3.1. Solubility

The solubility of myosin is closely linked to its functionality. Generally, the higher solubility of myosin enhances its functional properties during food processing [40]. The solubility of myosin is impacted by various factors, particularly the interactions between proteins and between proteins and water. Given the potential adverse health effects of high salt intake, researchers have begun to explore strategies involving BAAs to enhance myosin solubility at low salt concentrations, aiming to enhance the texture and tenderness.

Studies have shown that BAAs, when combined with NaCl, can significantly enhance myosin solubility and promote the dissociation of actomyosin. Fan, Gao, and Zhou [11] observed that the combination of 0.6% (*w*/*v*) L-Arg or 0.6% (*w*/*v*) L-Lys with 1.0% (*w*/*v*) NaCl not only effectively extracts myosin but also reduces the turbidity and particle size of myosin and actomyosin solutions while enhancing the residual activity of Ca^2^^+^. In addition, the antibacterial effect of NaCl can Mg^2^^+^-ATPase. These findings provide strong support for understanding how L-Arg and L-Lys, in combination with NaCl, improve the quality of meat products. Further research indicates that L-Lys and L-His can facilitate the transition of the α-helix structure to other secondary structures, exposing more sulfhydryl and hydrophobic groups, thereby further increasing myosin solubility. Guo, Peng, Zhang, Liu, and Cui [41] demonstrated that within the range of 0.01–0.6 mol NaCl, regardless of ion strength, L-Lys and L-His consistently improved the solubility of pork myosin, with L-Lys showing a more pronounced solubilizing effect. Moreover, the myosin tail region, rich in highly helical α-helix structures, tends to aggregate through electrostatic interactions between the rod-like tail regions at low ionic strengths, forming myosin filaments in water and diluting salt solutions [42]. However, the addition of L-His promotes the elongation of myosin light meromyosin and reduces the myosin filament formation ability by altering the charge redistribution in the myosin tail, thereby inhibiting filament assembly [43]. Furthermore, Chen et al. [44] found that the imidazole group in L-His disrupts hydrogen bonds within the α-helix due to its nucleophilic nature, altering intermolecular interactions and effectively inhibiting myosin filament assembly. This results in a decrease in protein particle size and an increase in the absolute value of ζ-potential, indicating that L-His enhances myosin solubility at low ionic strengths. L-Arg, on the other hand, promotes self-association through hydrogen bonds between the α-COOH, α-NH_2_, and guanidino groups of adjacent L-Arg residues, leading to increased aggregation and greater rigidity in the L-Arg side chains. The “gap effect” increases the activation energy required for polymer formation, thus reducing the rate of protein aggregation [45]. This explains why L-Arg, when bound to proteins, does not affect protein folding but effectively inhibits protein aggregation [46]. Shi et al. [47] further found that L-Arg, via its two -NH_2_ groups in the guanidine group, preferentially forms hydrogen bonds with the carbonyl oxygen atoms on the protein backbone, helping myosin resist aggregation under heat treatment. Moreover, BAAs can increase pH, thereby inhibiting the thermal aggregation of myosin and promoting protein solubility [47,48]. Based on these findings, BAAs improve myosin solubility by altering electrostatic and hydrogen bonding interactions.

### 3.2. Gel Properties

The gelation process of myosin is a complex transition from a sol to a gel state. Under heating conditions, myosin undergoes thermal denaturation, initially unfolding, and is possibly accompanied by reversible, unstable aggregation. As the temperature rises further, myosin gradually forms a more stable gel structure. During gelation, the key intermolecular forces involved include hydrophobic interactions, disulfide bonds, and hydrogen bonds. Under low-salt conditions, myosin molecules tend to aggregate, forming filament-like structures that are long and dense, resulting in the formation of chain-like thermogels. In contrast, at high salt concentrations, myosin predominantly exists in its monomeric form, crosslinking through both covalent and non-covalent bonds to form a 3D network structure [49].

In recent years, the use of BAAs has been explored to modify the gelation properties of meat proteins, finding that these amino acids can interact with myosin, altering its thermal aggregation behavior and thus improving gel performance. Research has shown that L-His, L-Lys, and L-Arg can influence the thermal gelation ability of chicken salt-soluble proteins under low ionic strength, enhancing gel properties [49,50,51]. BAAs enhance gel quality by increasing the number of hydrophobic residues and active sulfhydryl groups on the surface of salt-soluble proteins, modifying their molecular structure and thermal aggregation behavior. This results in gels with denser and more uniform structures [50,52]. Zhou, Li, and Tan [53] reported that adding 0.8% (*w*/*w*) L-Lys to pork sausage gel systems led to a denser and more uniform gel structure. In fish myosin systems, Gao, Wang, Mu, Shi, and Yuan [54] found that 5 mM L-His increased the solubility of myosin solutions from white bass under both low (0.1 M) and high (0.5 M) salt conditions. They suggested that L-His interacts electrostatically with the negatively charged surface of myosin, disrupting the protein’s electrostatic properties, altering the distribution of hydrophobic groups, and reducing hydrophobicity, thereby inhibiting intense protein aggregation during heating. Based on this, Gao et al. [55] showed that L-Arg exhibited better aggregation inhibition than L-His, and they explained that the pH increase resulting from BAAs is also essential to reduce protein aggregation [56]. Moreover, the special side-chain groups of L-Arg and L-His can selectively bind to aromatic amino acids exposed on proteins, slowing down protein denaturation and thus inhibiting aggregation, which favors the formation of superior gels. Man, Sun, Lin, Ren, and Li [38] investigated the impacts of L-Lys and L-Arg on the gel characteristics and molecular interactions of low-salt (NaCl, 1 g/100 g) mixed shrimp (*Antarctic krill* and *Pacific white shrimp*) surimi. The results showed that both L-Lys and L-Arg enhanced gel properties and WHC, outperforming gels with high salt (NaCl, 2.25 g/100 g). Meanwhile, among them, 0.3% L-Lys best improved the low-salt shrimp surimi gel’s hardness and springiness, increasing by 1.49-fold (770.43 g) and 1.17-fold (0.95), respectively, and its gel strength reached the highest value of 1676.56 g·mm, which was 378.83% higher than that of low-salt shrimp surimi gel. Molecular docking analysis further revealed that, in the low-salt gel system, L-Lys and L-Arg could stably bind to myosin, promoting the solubilization of MP and enhancing the interactions of hydrogen bonds and disulfide bonds, thus increasing the number of MP molecules involved in gel formation. Both L-Lys and L-Arg increased the proportion of myosin monomers, facilitating chain-like gel formation and improving water retention. Remarkably, L-Lys created a gel with clustered pores, while L-Arg resulted in a gel with a finer pore structure [49,51]. L-Arg promoted a continuous gel by changing actomyosin’s aggregation behavior, enhancing WHC and gel strength [50]. Thus, these findings suggest that BAAs interact with salt-soluble proteins and improve gel quality by affecting intermolecular interactions under low-salt conditions.

### 3.3. Emulsion Properties

The emulsifying activity and stability of muscle proteins are tightly connected with the quality and shelf life of emulsified meat products (like sausages). Myosin, as a key muscle protein, contains both hydrophilic and hydrophobic groups in its molecular structure. When myosin diffuses to the oil–water interface, its molecules unravel and rearrange to form a tightly packed interfacial protein membrane [57]. As an amphiphilic molecule, myosin theoretically exhibits good emulsifying properties, forming a mechanically stable interfacial film at the oil–water (O/W) interface. This film prevents the aggregation of oil droplets via electrostatic forces and spatial interference [58]. Studies have suggested that BAAs can improve protein emulsifying properties in systems like sausages via two main mechanisms: (1) BAAs, as amphiphilic molecules, directly enhance emulsification [57], and (2) they indirectly enhance emulsification by altering protein structure [59].

When ground chicken breast meat was emulsified with BAAs at a 1% NaCl concentration, it led to the formation of emulsion droplets with smaller and more uniform sizes [60]. In a MP–soybean oil emulsion system, Zhu, Li, Li, Ning, and Zhou [59] discovered that the addition of 0.1% (*w*/*w*) L-Lys and L-Arg led to a reduction in droplet size, a decrease in viscosity, and an increase in the absolute value of the ζ-potential after high-speed shearing. Compared with the control group, the emulsifying activity indices in the groups with 0.1% (*w*/*w*) L-Lys and L-Arg increased by 3.59% and 3.33%, respectively. They also observed the accumulation of L-Lys and L-Arg on the oil droplet surface, indicating that these amino acids, as amphiphilic molecules, not only adsorb at the oil–water interface but also enhance O/W emulsion stability. However, when the addition of L-Lys and L-Arg increased to 0.3% (*w*/*w*), emulsion stability decreased, suggesting that at this concentration, their inhibition of protein aggregation was weaker than the interactions between droplets [61]. The effects of different amino acids on myosin emulsification properties vary. L-Arg increases myosin’s interfacial pressure and penetration rate while decreasing its diffusion rate, whereas L-Lys enhances the penetration rate but reduces both interfacial pressure and diffusion rate. Additionally, L-Arg favors the ordered arrangement of myosin at the interface, while L-Lys tends to expose tyrosine and tryptophan residues of myosin at the interface.

Research has also explored how BAAs improve emulsification by altering protein conformation. BAAs induce structural transitions in interfacial myosin, enhancing interactions between the oil phase and myosin and ultimately improving emulsion stability [62,63]. Similar phenomena were observed in low-ionic-strength porcine MP emulsions, where at pH 6.5, adding 1 g/L of L-His or L-Lys to MPs solutions (10 mg/mL) mixed with soybean oil at a 4:1 (*v*/*v*) ratio improved emulsifying activity, reduced emulsion index and droplet size, enhanced protein diffusion, and increased interfacial pressure. Raman spectroscopy analysis further indicated that L-His, L-Lys, and L-Arg caused interfacial MP to unfold, facilitating the transition of the α-helix to other secondary structures, thus enhancing the stability of the system under low-ionic-strength conditions [64]. Li et al. [57] also reported that these structural changes, particularly the transformation of random coils into ordered secondary structures, promoted the formation of a dense, mechanically strong interfacial membrane. In the research on porcine myosin aggregation behavior, Han et al. [65] found that the changes in secondary structure and the exposure of hydrophobic binding sites could influence the aggregation patterns and microstructures of emulsified gels. Ban et al. [39] reported that BAAs induce the exposure of aromatic and hydrophobic amino acids, altering the rheological and structural properties of emulsified gels.

**Figure 4:**
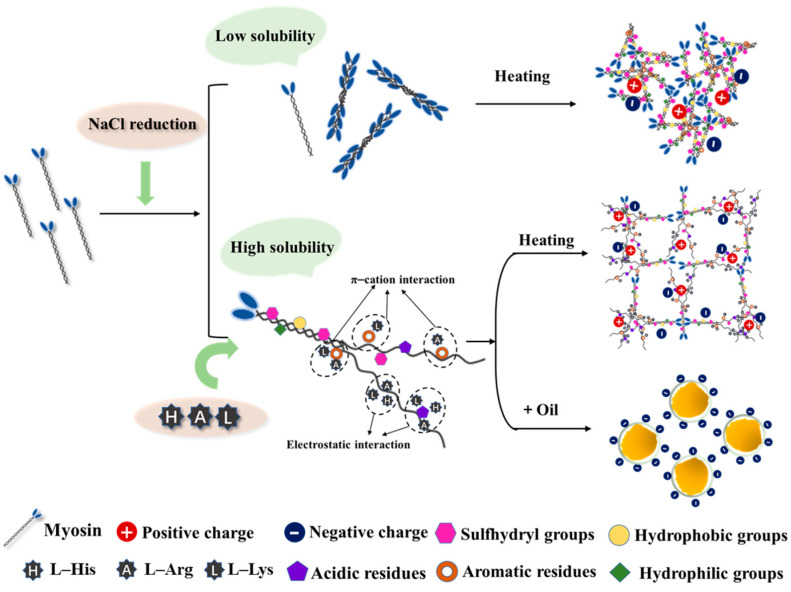
Effect of BAAs on processing of low-salt meat products.

As shown in Figure 4, we propose a mechanism in which L-His, L-Lys, and L-Arg influence myosin aggregation. Under low-salt conditions (<0.3 mol/L), myosin forms a fragmented, porous network structure due to its relatively low solubility. In such a situation, BAAs interact with myosin’s aromatic residues via cation–π and electrostatic interactions. This unfolds the myosin α-helix, exposing hydrophobic tryptophan, tyrosine, and sulfhydryl groups, enhancing solubility. These changes promote myosin–myosin and myosin–water interactions when heated, improving gelation. Furthermore, they enhance myosin adsorption at the oil–water interface, enhancing the protein membrane, reducing interfacial tension, and improving emulsion stability.

## 4. The Effect of BAA on Low-Salt Gel-Based Meat Processing

### 4.1. Protein and Lipid Oxidation

In meat product processing, salt addition significantly affects flavor and texture and can also alter protein structure and oxidation state by modifying ionic strength. NaCl, a common oxidant promoter, enhances protein sensitivity to oxidants by adjusting ionic strength, thus influencing protein carbonylation [66]. Moderate protein oxidation can strengthen intermolecular interactions, promote cross-linking, and improve gel texture [59,67]. However, excessive oxidation may destroy product quality [68]. Therefore, controlling oxidation levels is critical in processing low-salt gel-type meat products. Previous reports have studied the impacts of BAAs on protein and lipid oxidation during the processing of low-salt meat products. The potential mechanisms are shown in Table 1.

BAAs have been shown to significantly influence the oxidative behavior of meat proteins. L-Arg and L-Lys promote the transition of the α-helix structure to the β-sheet structure. Specifically, L-Arg and L-Lys facilitate the formation of disulfide bonds, effectively inhibiting the excessive aggregation of MPs, alleviating the decrease in myosin heavy chain (MHC) density, and reducing the oxidation of polar amino acids in MPs [12]. In a study on the impact of NaCl replacement on protein and lipid oxidation in Harbin red sausage, Wen et al. [69] found that compared to the 100% NaCl group, replacing 30% NaCl with a mixture of 4% L-Lys, 20% KCl, 0.5% citric acid, 1% L-Arg, 3.5% maltodextrin, and 1% Ca-lactate reduced carbonyl content. After adding 1.25% NaCl with L-Lys (0.2%, 0.4%, and 0.6%), the sulfhydryl group content in restructured ham was higher than in the group with only 2.5% NaCl. This suggests that BAAs can reduce sulfhydryl group loss, delaying protein oxidation in reduced-salt meat products [10]. Additionally, BAAs possess the ability to scavenge hydroxyl radicals (·OH) and chelate ferrous ions, thereby delaying the oxidation of meat proteins [53]. They exhibit intrinsic antioxidant activity, with L-Arg, for example, reacting with reactive oxygen species (ROS) via its guanidino group, converting them into more stable products and thus reducing the attack of hydroxyl radicals on proteins, as shown in Table 1 (Equation (1)). Metal ions (such as iron and copper) act as catalysts in protein oxidation, facilitating ROS generation or directly participating in oxidation reactions. BAAs can bind these metal ions, inhibiting protein oxidation. The imidazole group in L-His has a strong affinity for metal ions and can form stable complexes with iron (Fe^2^^+^ or Fe^3^^+^), rendering the metal ions catalytically inactive, as illustrated in Table 1 (Equation (2)). L-Lys, L-Arg, and L-His can competitively bind with ·OH radicals, leading to the production of 2-amino-adipic semialdehyde, 2-oxo-histidine, and glutamic semialdehyde, respectively, thereby inhibiting protein oxidation by these radicals [70,71]. Furthermore, Xu et al. [72] demonstrated that increasing L-Lys and L-Arg effectively scavenged DPPH radicals, reduced carbonyl content under OH-induced oxidative stress, and increased the chelation rate with ferrous ions. This inhibited the propagation of free radical chain reactions. While this cannot entirely eliminate the negative effects of oxidation, it can significantly delay protein oxidation in meat [73].

In salted meat processing, the effect of NaCl on lipolysis has long been a research focus. When Choi studied the effect of NaCl on lipid oxidation in oil-in-water emulsions, he found that a study showed adding 1.0% NaCl could reduce lipid oxidation with sodium dodecyl sulfate (SDS)-stabilized by 20% [74]. Research revealed that NaCl affected lipid oxidation kinetics by altering the critical micelle concentration (CMC) of SDS in O/W emulsions. This changed the distribution of hydroperoxides (LOOH) and the antioxidant δ-tocopherol, thus influencing lipid oxidation [75]. In addition, a 1% substitution of KCl for NaCl led to a significant alteration in the CMC of CTAB. But when the KCl concentration reached 171 mM, which was equivalent in molarity to 1% NaCl, the impact on the CMC became rather insignificant. From a mass-based perspective, replacing NaCl with KCl influenced the formation of lipid hydroperoxides in emulsions. It achieved this by decreasing the amount of emulsifier micelles present in the aqueous phase [76]. Up to now, the influence of replacing NaCl with KCl on the oxidation of lipids has been mainly documented in meat-related research.

Meanwhile, in recent years, the exploration of alternative substances to regulate lipid oxidation in meat products has become another research hotspot. Among them, BAAs have emerged as a promising option, showing significant potential in inhibiting lipid oxidation.

Lipid oxidation plays a crucial role in the degradation of meat product quality, producing both primary and secondary oxidation products that negatively impact flavor and texture. NaCl is known to accelerate triglyceride oxidation, potentially by displacing iron ions [77]. Recent studies have indicated that BAAs can inhibit lipid oxidation [78]. Liu et al. [79] found that substituting 30% NaCl with 18% potassium lactate and 12% L-Lys in the curing of ham markedly elevated peroxide value and thiobarbituric acid reactive substances while also enhancing the activity of acidic lipase, neutral lipase, and phospholipase, as well as the content of free fatty acids, which contrasted with the full-salt group. This suggests that BAAs can promote flavor development in ham by influencing lipid oxidation and hydrolysis. In addition to scavenging free radicals and chelating metal ions, BAAs can react with primary oxidation products such as aldehydes and ketones, which are major contributors to flavor degradation and quality loss in meat products [80]. Deng et al. [81] reported that compared to 3% NaCl, 1% L-Arg and L-Lys significantly reduced lipid oxidation in pan-fried beef patties at 1% NaCl concentration. The guanidino group of L-Arg donates hydrogen atoms to peroxy radicals, converting them into more stable products and preventing further lipid oxidation, as shown in Table 1 (Equation (3)). The imidazole group in L-His strongly binds to metal ions, forming stable complexes with iron (Fe^2^^+^ or Fe^3^^+^) and inhibiting the catalytic activity of iron in lipid oxidation, as shown in Table 1 (Equation (4)). L-Lys can react with aldehydes generated during lipid oxidation to form Schiff bases, reducing the harmful impacts of aldehydes on meat product flavor and acceptability, as shown in Table 1 (Equation (5)). These BAAs effectively inhibit lipid oxidation [82].

BAAs exert antioxidant effects through various mechanisms, including the scavenging of reactive oxygen species, chelation of metal ions, and reactions with lipid oxidation products. These actions effectively delay the oxidation process in meat products, thereby improving their quality and flavor. Qiu [83] found that 0.25% NaCl + 0.1% L-Arg had a saltiness between 0.25% and 0.5% NaCl, showing L-Arg’s salt-compensating effect. An electronic-tongue comparison revealed that arginine reduced-sodium salt was saltier than non-iodized reduced-sodium salt but close to seaweed-iodine salt. However, the actual inhibitory effect depends on factors such as the specific type of meat product, the processing methods, and the type and concentration of the BAAs used.

### 4.2. Sensory Properties

The use of BAAs in low-sodium gel-based meat products has emerged as an important area of research in recent years, particularly for improving flavor and texture and reducing salt content. Table 2 outlines the impact of substituting NaCl with BAAs on the sensory and texture properties and harmful substances in low-salt gel-based meat products.

Studies have shown that BAAs significantly enrich the flavor of low-salt meat products. Wang, Luo, Guo, Wang, and Xia [36] demonstrated that a 15 mM L-Lys-assisted water-bath microwave heating (L-WB40 + MW10) treatment of fish surimi allowed for a decrease in salt content without affecting the texture or saltiness of the surimi gel. These amino acids improve umami flavor and reduce undesirable odors through interactions with chloride salts, thereby enhancing overall sensory quality, despite having relatively weak intrinsic flavors [92]. In Bologna sausage prepared with 1% NaCl, 1.5% KCl, 1% L-Arg, and 0.2% L-His, a 40% reduction in sodium content was achieved while maintaining high sensory performance [84]. Similarly, Wang, Cui, Zhang, Hayat, and Ho [93] reported that adding 0.4% Amadori rearrangement products enabled a 20% reduction in NaCl while significantly enhancing umami attributes. Wang [94] compared the effects of five amino acids (L-Lys, L-Arg, L-His, L-Pro, and Glycine) on the solubility and gel characteristics of MP systems. The study found that 15 mmol/L of L-Lys or 20 mmol/L of L-Arg were most effective in low-sodium gels, increasing MP solubility and inhibiting excessive protein aggregation. This promoted the development of a continuous 3D network structure, enhancing gel WHC. Furthermore, L-Lys co-used with water-bath microwave heating in fish surimi decreased MHC degradation, increased disulfide bond formation, and promoted ε-(γ-Gln)-Lys covalent crosslinking, resulting in a 15% salt reduction while preserving the perception of saltiness. Several studies have shown that replacing NaCl with BAAs can mitigate the off-flavors caused by partial NaCl substitution. Campagnol, Santos, Morgano, Terra, and Pollonio [85] reported that substituting 50% NaCl with L-Lys and KCl in pork sausage alleviated the undesirable odors resulting from KCl and enhanced the sausage’s texture and color. In another study, Vidal, Santana, Paglarini, Silva, and Pollonio [86] demonstrated that adding 3% L-Lys to a low-sodium cured meat formulation (50% NaCl, 25% KCl, and 25% CaCl_2_) significantly improved the entire acceptability of products. Likewise, Silva et al. [84] observed similar results. Meanwhile, L-Arg’s salt-compensating effect is not via the ENaC channel, and its salt-perception-enhancing pathway needs further study [83].

Thus, the interactions between BAAs and chloride enhance the saltiness and flavor profile of low-salt gel-based meat products, making BAAs a promising ingredient for developing such products. However, compared to the extensive research on the gelation and emulsifying properties of BAAs, studies on their mechanisms in sensory enhancement remain limited. It is anticipated that, in the near future, researchers will focus on understanding the sensory pathways of amino acids and the interaction mechanisms between BAAs and additional components.

### 4.3. Textural Properties

During meat processing, NaCl is commonly used as an additive. The ionic shielding effect resulting from its dissociation significantly promotes the solubility of salt-soluble proteins, reducing electrostatic repulsion between molecules. This facilitates protein unfolding and the stable three-dimensional gel network creation via hydrophobic interactions, hydrogen bonding, and disulfide cross-linking. This gel structure is crucial for the gelation quality of meat products. NaCl not only alters protein conformation, enhancing the binding of hydrophilic groups with water molecules but also effectively binds water in the gel network, maintaining the juiciness and tenderness of the product. However, with the growing trend toward reduced-salt and low-sodium meat products, reducing NaCl content may negatively impact product quality. Alio, Fuentes, Fernández-Segovia, and Barat [95] successfully prepared low-sodium cod products with a 25% KCl + 75% NaCl (*w*/*w*) mixture, maintaining high consumer acceptance and suggesting that KCl can partially replace NaCl to preserve sensory attributes. Additionally, several studies also provided evidence that divalent cations could cause excessive unfolding of the protein structure. This decreased WHC and led to the formation of a coarse and disordered three-dimensional gel network structure [96,97]. This outcome may result from the lower ionic strength of CaCl_2_ or MgCl_2_, which weakens the solubility of proteins, thus reducing emulsifying stability and cooking yield [98,99].

Thus, BAAs in low-salt gel-based meat products have garnered increasing attention. Zhang et al. [60] found that the impacts of BAAs on chicken breast acceptability were investigated at a 1% NaCl level. The results showed that adding 0.06% BAAs significantly improved the myofibril fragmentation, MP solubility, emulsion activity, storage modulus rate, WHC, and hardness when compared with the 1% NaCl-only group. The effects varied with different types of BAAs but their positive influence on the quality of low-salt meat products, especially with lower NaCl content, was evident. Jiang et al. [87] found that in low-salt whiteleg shrimp subjected to microbial transglutaminase (MTGase), the addition of L-Arg significantly enhanced gel properties in spite of a low concentration (0.5%). Similar effects were observed in low-salt Chinese shrimp surimi, as reported by Liu et al. [89], who studied the impact of L-Arg on low-salt (0.5% NaCl) Chinese shrimp surimi gel (SSG). Gel strength and hardness significantly increased as the concentration of L-Arg rose from 0 to 0.75%, outperforming the high-salt (2% NaCl) samples. The study concluded that 0.75% L-Arg improved protein solubilization (up to 74.89%) and hydrogen–disulfide bonds, resulting in a continuous gel structure and improved gel properties. Furthermore, L-His was shown to covalently bind with epigallocatechin-3-gallate (EGCG) via Michael addition or Schiff base reactions, enhancing the antioxidant capacity of meat proteins (such as MP). This interaction reduced gel cooking loss (from 66.7 ± 0.23% to 40.3 ± 2.02%) and improved rheological properties, with gel strength increasing from 0.10 ± 0.01 N to 0.22 ± 0.03 N. Thus, L-His–EGCG promotes the creation of a dense and continuous gel structure, enhancing WHC [88]. Thus, it is apparent that BAAs significantly enhance gel quality under low-salt conditions, offering a promising approach to enhancing the palatability of gel-based meat products.

### 4.4. Influence of BAA on Harmful Substances in Low-Salt Meat

During meat processing, BAAs demonstrate significant inhibitory effects on harmful substances. Studies have shown that various levels (0.1%, 0.5%, 1.0%) of BAAs significantly reduce the creation of total heterocyclic amines (HCAs) in beef patties treated with 1% NaCl, with 1% L-Lys exhibiting the most effective inhibition. L-Lys reduced total HCA content to 2.46 ng/g, achieving a 70.88% inhibition rate [81]. Moreover, L-Arg, L-His, and L-Lys all significantly inhibited the formation of β-carbolines, with L-His showing the best performance in roasted beef patties. L-His demonstrated excellent scavenging ability for alkyl radicals and could competitively inhibit tryptophan, making it a promising alternative to synthetic antioxidants, with added benefits of lower cost and higher safety. This makes L-His a viable option for reducing carcinogenic compounds in meat and fish products. Additionally, Linghu, Karim, and Smith [91] pointed out that L-Lys can inhibit the formation of 2-amino-1-methyl-6-phenylimidazo [4,5-b] pyridine (PhIP) by scavenging phenylacetaldehyde. Xue et al. [90] found that L-His suppresses the generation of norharman and harman in beef patties and model systems by scavenging free radicals and competing with tryptophan. These findings highlight the significant potential of BAAs in inhibiting the formation of harmful substances at low NaCl concentrations, which has important implications for enhancing meat product safety. However, despite research on the impacts of BAAs on gelation and emulsification in meat products, studies on their mechanisms for controlling harmful substances in low-salt meat processing remain limited and warrant further investigation.

## 5. Future Perspectives

With the growing awareness of health risks linked to high-sodium diets, the demand for low-sodium meat products has sharply increased. BAAs, as safe and effective additives, enhance the gelation properties and sensory acceptance of low-sodium gel-type meat products, offering significant potential for their application. Recent studies have focused on salt reduction technologies, such as ultrasound and pulsed electric fields, which facilitate the diffusion and release of sodium ions, enabling salt reduction without compromising perceived saltiness [34,85,100]. Additionally, the combination of L-Lys and microwave heating has been shown to improve gel strength and promote the network in low-sodium fish paste, effectively retaining moisture [36]. Integrating BAAs with these physical methods holds promise for further enhancing the quality and consumer acceptance of low-sodium meat products.

Because of differences in muscle composition and types, the structure and function of salt-soluble proteins vary significantly. While it is established that BAAs can improve the solubility and gelation characteristics of salt-soluble proteins, such as myosin, the precise mechanisms remain unclear. To fully grasp the molecular interactions between BAAs and key salt-soluble proteins, future studies could utilize high-resolution structural techniques, including nuclear magnetic resonance (NMR) and small-angle X-ray scattering (SAXS), to explore how BAAs induce protein conformational changes and affect functional properties, such as enzymatic activity and biomolecular interactions. In addition, advanced characterization methods, such as atomic force microscopy (AFM) and confocal laser scanning microscopy (CLSM), could be employed to investigate the structural formation mechanisms of BAAs in low-salt gel-type meat products. This will provide insights into protein aggregation, crosslinking, and interactions with other ingredients, contributing to dynamic models of multi-scale structural formation.

Moreover, combining modern food processing with big data and AI, based on consumer preferences, can help develop custom production models for low-salt gel meat products. This allows for the precise control of the gel structure and process optimization, providing support for creating healthier, customized low-salt meat products. BAAs used in salt-reduced gel meat products have complex economic implications. In the long run, BAAs can improve the gel properties and sensory quality of low-sodium meat products, and reduce product scrap and waste caused by poor quality. If more accurate addition and process control can be achieved, it may improve production efficiency and shorten the production cycle, thus saving costs to a certain extent. Promoting the upgrading and development of the entire meat processing industry has important economic significance and potential.

## Figures and Tables

**Table 1:** The potential mechanism of BAAs on meat protein and lipid oxidation.

**Type**	**Equations**	
Protein oxidation	$L-Arg-NH-C({NH}_{2}{)}_{2}+\cdot OH\to H_{2}O+L-Arg-NH-C(NH\cdot ){NH}_{2}$	(1)
	$nHis+{Fe}^{n+}\to [Fe(His{)}_{n}{]}^{n+}$	(2)
Lipid oxidation	$L-Arg-NH-C({NH}_{2}{)}_{2}+ROO\to ROOH+L-Arg-NH-C\left(NH\cdot \right)NH_{2}$	(3)
	${nHis+Fe}^{2+}\to [Fe(His{)}_{n}{]}^{2+}$	(4)
	$Lys-NH_{2}+R-CHO\to Lys-N=CH-R+H_{2}O$	(5)

Note: in the reactions, *n* represents the number of L-His molecules.

**Table 2:** Effects of BAA partial substitution of NaCl on texture and sensory properties of meat products and harmful substances during processing.

Product	Salt Mixture	Mechanism	Result	**Reference**
Harbin dry sausage	70% NaCl, 20% KCl, 4% L-Lys, 1% L-Arg, 0.5% citric acid, 1% Ca-lactate, and 3.5% maltodextrin	Enhancing the formation of volatile compounds from carbohydrate and amino acid metabolism, β-lipid oxidation, and esterification.	Improving the flavor and reducing NaCl by 30%.	[69]
Pork emulsion sausages	2.2 g of low-sodium salt + 0.6 g L-Arg and L-Lys	L-Arg and L-Lys could retard the total SH content reduction.	L-Arg and L-Lys could retard meat proteins form oxidation under ·OH stress.	[73]
Low-fat bologna sausages	1% NaCl + 1.5% KCl + 1% L-Arg + 0.2% L-His	Increasing the pH values and deviating from the isoelectric point of MP via the formation of hydrogen bonds and ion–dipole interactions between the side chains of these amino acids and water.	Producing products with a 40% sodium reduction while ensuring adequate processing and sensory properties.	[84]
Fermented cooked sausages	0.313% L-Lys and a mixture of taurine (750 mg/kg) with disodium inosinate (300 mg/kg) and disodium guanylate (300 mg/kg)	The pH decreased significantly, while Aw ranged from 0.905 to 0.916, showing no significant difference between the modified products and the control.	Improving the flavor issues due to KCl.	[85]
Salted meat	50% NaCl, 25% KCl, 25% CaCl_2_ + 3% L-Lys	Decreasing moisture content.	Minimizing the negative sensory impact of KCl and CaCl_2_, decreasing the salty taste and aftertaste in mixed-salt meat products, without affecting physicochemical quality parameters.	[86]
Ground chicken breast meat	1% NaCl + 0.06% L-Arg, L-Lys, and L-His (*w*/*w*)	Increasing pH, WHC, and solubility.	Compared to the 1% NaCl (*w*/*w*) treatment, adding 0.06% BAAs (*w*/*w*) significantly increased MP solubility, emulsion activity, storage modulus change rate, gel WHC, and hardness.	[60]
Reduced-salt whiteleg shrimp surimi	0.5% NaCl, 0.75% MTGase, and different contents (0.5%, 1%, 1.5%, 2.0%, and 2.5%) of L-Arg	Adding L-Arg and MTGase together significantly increased disulfide bonds and the protein β-sheet structure of SSG, while enhancing moisture distribution and rheological properties.	Combining L-Arg and MTGase improved the gel characteristics of SSG while reducing NaCl content.	[87]
Pork meat	EGCG–His complex at a molar ratio of 1:5	Forming an EGCG–Histidine complex through covalent binding of histidine to EGCG via Michael addition or Schiff base reaction significantly increases the antioxidant activity of the complex compared to EGCG or histidine alone.	Decreasing cooking loss (40.3 ± 2.02%), enhancing rheological properties, and enhancing gel strength (0.22 ± 0.03 N) of MP.	[88]
Chinese shrimp surimi	0.75% L-Arg (*w*/*w*)	Enhancing protein solubility, hydrogen bonds, and disulfide bonds in SSG, L-Arg addition resulted in a denser network structure, as observed by Cryo-SEM. Molecular docking revealed L-Arg interaction with myosin through hydrogen bonds, significantly increasing protein solubility to 74.89%.	Increasing protein solubility, hydrogen bonds, and disulfide bonds with 0.75% L-Arg, forming a denser gel network structure for low-salt SSG, thereby improving gel properties.	[89]
Roast beef patties	0.1%, 0.5%, 1.0% L-His (*w*/*w*)	Attributing the inhibitory mechanism of L-His to free radical scavenging and competitive inhibition.	Demonstrating excellent alkyl radical scavenging ability, with a maximum of 82.59%, L-His effectively reduced radical activity.	[90]
A model system consisting of reducing sugars, creatinine, and phenylalanine to investigate PhIP formation	Reactivity of each AA to the total PheAce in PheAce-containing model system, wherein 0.04 mmol PheAce used as precursor reacted with AA solution (0.4 M) in final molar ratio of PheAce:AA at 1:0, 1:0.125, 1:0.25, 1:0.5, 1:1, and 1:2.	Scavenging HAs directly by L-Lys and forming adducts with L-Lys, which may contribute to reducing HA content.	Inhibiting the aldol condensation between creatinine and phenylacetaldehyde to form PhIP, phenylacetaldehyde-Lys adducts in the Maillard reaction reduced PhIP content in the final product in a dose-dependent manner.	[91]
Emulsion sausage	0.4% L-Arg and L-Lys (*w*/*w*)	Sharing a similar molecular structure with L-Lys and L-Arg exhibits comparable capacity to scavenge free radicals.	Effectively inhibiting lipid and protein oxidation in emulsion sausage, both L-Lys and L-Arg scavenged free radicals and chelated ferrous ions.	[72]
Fried beef patties	1% NaCl + L-Arg, L-Lys, and L-His (0.1%, 0.5%, 1%, *w*/*w*)	-	The addition of BAAs at 1% NaCl significantly enhanced the quality characteristics compared to 3% NaCl.	[81]

## Data Availability

No new data were created or analyzed in this study.
